# Systematic review of the efficacy of a hybrid operating theatre in the management of severe trauma

**DOI:** 10.1186/s13017-021-00390-z

**Published:** 2021-08-28

**Authors:** Chun Yuet Khoo, Terence Yi Song Liew, Sachin Mathur

**Affiliations:** Department of General Surgery, Singapore General Hospital, 20 College Road, Academia, Singapore, 169856 Singapore

**Keywords:** Hybrid OT, Hybrid operating theatre, Trauma, Injury, Interventional radiology

## Abstract

**Background:**

Hybrid operating theatres (OT) allow for simultaneous interventional radiology and operative procedures, serving as a one-stop facility for the treatment of severely injured patients. Several countries have adopted the use of the hybrid OT however their clinical impact in improving efficiency and quality of care remains unclear. This study systematically reviews the clinical impact of the hybrid OT for treatment of the severely injured.

**Methods:**

A literature review of the PubMed, Embase and Cochrane databases was performed to identify all published articles in English, from 1st January 2000 to 31st December 2020, reporting on the impact of a hybrid OT for severe trauma. Articles were also reviewed for references of interest.

**Results:**

Five studies reporting the clinical impact of the hybrid OT, in a total of 951 patients, were shortlisted. All were cohort studies that compared patient outcomes in the hybrid OT versus a conventional group. Out of 3 studies that assessed timeliness to intervention, one reported shorter time associated with the hybrid OT, while the other two reported no difference. Mortality outcomes were reported in 4 studies and showed no significant difference associated with treatment in the hybrid OT. Two studies revealed shorter total procedure times associated with the hybrid OT. Two out of 3 studies that evaluated blood transfusion requirements reported decreased transfusion rates in the hybrid OT group. Only 1 study examined complication rates and demonstrated morbidity benefits associated with the hybrid OT.

**Conclusion:**

Establishment of a hybrid OT requires a significant capital investment as well as a highly functioning multi-disciplinary team. The cost–benefit ratio remains unclear. Future studies, preferably in the form of clinical trials, are required to evaluate its usefulness in improving timeliness to definitive haemorrhage control and outcomes in severe trauma.

## Introduction

Despite many advances in trauma management, haemorrhage still remains the most common cause of preventable mortality in severely injured patients [1, 2]. Clarke et al. reported that every 3-min delay to the operating theatre (OT) for laparotomy increased mortality by 1% [3]. Rapid access to the OT, interventional radiology (IR) or a combination of both is required to reduce this mortality overall.

Hemodynamically unstable patients assessed in the emergency department (ED) will not usually progress for a computed tomography (CT) scan and may require separate transfers to IR or OT for definitive haemorrhage control. On the other hand, hybrid OTs may allow for simultaneous IR and operative procedures, serving as a one-stop facility to reduce intra-hospital transfers and potentially shorten the time to definitive treatment. In 2013 it was noted that there was significant potential to expedite haemorrhage control with this new treatment paradigm and clear guidelines regarding patient selection and justification for cost expenditure were required [4].

Since then, many countries have added the hybrid OT to their infrastructure planning but the clinical impact in terms of achieving expedient care and reducing mortality in major trauma remains unclear. Furthermore, reviews of the hybrid OT setup have been limited to cardiothoracic, orthopaedic, vascular and neurosurgery disciplines rather than trauma [5]. In this systematic review we aimed to consolidate all recently published studies reporting on the impact of a hybrid OT for severe trauma and discuss its utility.

## Methods

### Search strategy and study identification

A systematic literature search was performed in accordance with Preferred Reporting Items for Systematic Reviews and Meta-Analyses (PRISMA) guidelines. A comprehensive search of the PubMed, EMBASE and Cochrane Central Register of Controlled Trials databases yielded 5758 papers published in English between 1 January 2000 and 31 December 2020. For the PubMed search, the MeSH term “operating rooms” was used. Keywords used include “hybrid operating theatre”, “hybrid operating room”, “hybrid OR”, “operating theatre”, “operating room”, “OR”, “angioembolization”, “interventional radiology”, “trauma” and “injury”. For the EMBASE search, the same keywords were used in combination with the emtree term “injury”. For the Cochrane database search, the same keywords were used in combination with the emtree terms “operating rooms”, “interventional radiology” and “wounds and injuries”. Attempts were made to search the grey literature using Google search engine. Title/abstract screening were performed independently by two study investigators (CK/TY) to identify articles of interest. The final search result of eligible articles were discussed with the senior author (SM). All retrieved publications and their references were manually reviewed.

### Study selection criteria and eligibility criteria

The inclusion criteria were (1) English-language studies, (2) full-text articles, (3) articles that evaluated the impact of a hybrid operating theatre for severe trauma. The exclusion criteria included (1) abstracts, letters, editorials, expert opinions, technical notes, case reports and reviews, (2) articles written in a language other than English.

### Data extraction and statistical analyses

Data obtained from the full-text articles included: year published, country, study cohort, interventions performed, study outcomes and follow-up duration. The primary outcomes of interest were time to intervention and overall mortality. Additional outcomes include total procedure time, blood transfusion requirements and in-hospital complications. The Newcastle–Ottawa Scale for assessing risk of bias for cohort studies was independently applied by two investigators (CK/TL) to assess the quality of the studies reviewed. Any disagreement was resolved by discussion with the senior author (SM).

A meta-analysis was not feasible as the five shortlisted studies had significant heterogeneity in terms of study populations, type of interventions compared and measures of outcome. The small number of studies and the small sample size of each study also compromised the quality and possibility of a meta-analysis. Hence, no statistical analysis was performed. Therefore, a narrative approach was adopted to appraise the utility of the hybrid OT based on reported data so as to generate hypotheses and impetus for further research.

## Results

### Search results

**Fig. 1:**
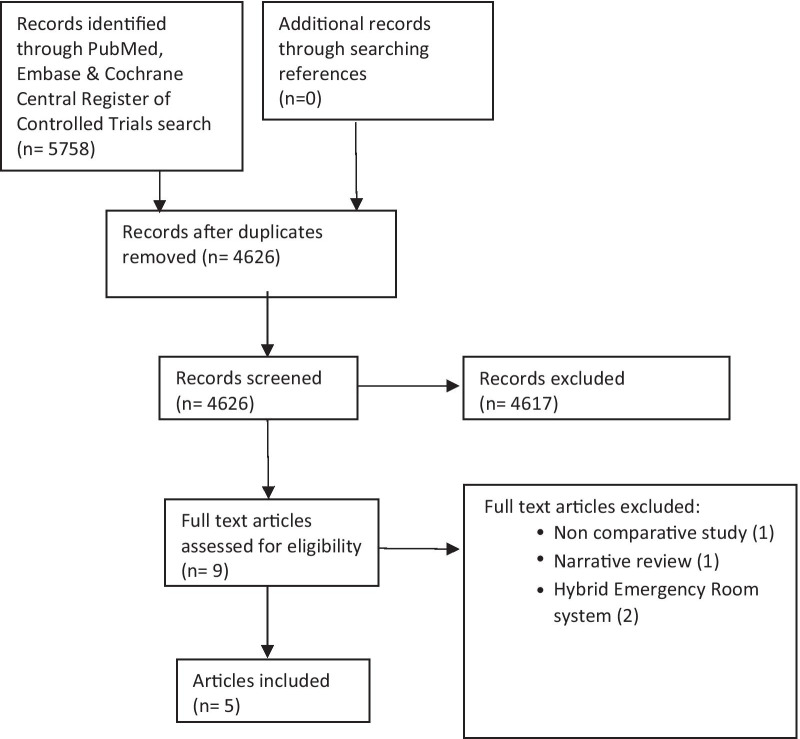
PRISMA flow diagram of literature search

The abstraction process is summarised in Fig. 1. The PubMed search yielded 1804 records, Embase 3873 and the Cochrane database 81. Overall 1132 duplicate articles were excluded and 4626 articles underwent abstract review. Nine potential articles that evaluated a hybrid OT setup for trauma patients underwent full-text evaluation. Of these, 5 articles fulfilled our inclusion and exclusion criteria and were included in this review. No additional articles were identified from the reference list of these studies.

### Hybrid OT characteristics

Each study represented a different jurisdiction (Switzerland, USA, Canada, Japan, Korea) and described a standalone hybrid OT, situated in proximity to the ED. One setup was described as a RAPTOR suite (Resuscitation with Angiography, Percutaneous Techniques and Operative Repair) [6], though all studies described mobile C-arm and angiography capabilities. Gross et al. described a unique multi-functional image guided therapy suite (MIGTS) that included a CT scanner within the hybrid OT adjacent to the main operating theatre table [7]. Four of the 5 hybrid OT suites were available to trauma patients 24/7; only Kataoka et al. had limited capabilities (daytime, weekdays) [9]. Three studies described the cost of the hybrid OT setup between $1.5million (Loftus, Kataoka et al.) and $3.6million [€3million] [7–9].

All five shortlisted studies were cohort studies with no randomised controlled trials, presenting data for 951 patients who underwent interventions (angiographic procedures and/or surgery) for traumatic injuries in either the hybrid OT (*n* = 481) or non-hybrid setup (*n* = 470) (Table 1) [6–10]. There was significant heterogeneity between each study with regards to methodology for cohort comparison. The 2 prospective studies differed as Gross et al. divided the cohort by the availability of the MIGTS at the time of the major trauma presentation, whereas Carver et al. admitted all trauma activated patients to the RAPTOR suite hence comparison was made with a retrospective pre-RAPTOR cohort [6, 7]. Within the retrospective studies, Loftus et al. considered patients who underwent first procedure within 4 h, whereas Jang et al. only considered patients who underwent first OT or IR procedure within 2 h [8, 10].

**Table 1:** Characteristics of the five included studies which evaluated the efficacy of a hybrid operating theatre for severe trauma

Author, Year	Country	Study Method	Hybrid OT concept	Hybrid OT group (number)	Conventional group (number)	Study population	Interventions	**Follow-up**
Gross, 2009	Switzerland	Prospective cohort that underwent either hybrid or conventional treatment based on availability of the MIGTS	MIGTS (hybrid OT incorporating CT scanner)	87	81	Mean 42 years old, Consecutive multiply injured trauma patients (ISS > / = 16)	Diagnostic CT scan, angiographic procedures and/or surgery	24 months
Loftus, 2020	USA	Retrospective cohort comparing before (conventional group) & after (hybrid OT) implementation of a hybrid OT	Remodeled angiography suite within an OT	186	106	Median 41 years old, Consecutive adult trauma patients who underwent immediate surgery (within 4 h of arrival at a trauma center)	Angiographic procedures and/or surgery	In-hospital stay
Carver, 2020	Canada	Prospective cohort (hybrid OT group) compared with a consecutive retrospective cohort (conventional group)	RAPTOR suite	169	169	Mean 38 years old, Adult trauma patients severely injured (ISS > / = 12) and required an intervention	Angiographic procedures and/or surgery	In-hospital stay
Kataoka, 2016	Japan	Retrospective cohort that underwent either hybrid or conventional treatment within the same period	Remodeled angiography suite within an OT	13	45	Age > / = 16 years old, Severely injured patients (ISS > 15) who underwent both emergency surgery and IR	Angiographic procedures and surgery	In-hospital stay
Jang, 2020	Korea	Retrospective cohort that underwent either hybrid or conventional treatment within the same period	Remodeled angiography suite within an OT	26	69	Median 54 years old, Trauma patients (ISS > / = 9) who underwent emergency surgery and/or IR within 2 h on admission	Hybrid group: Angiographic procedures OR combined angiographic procedures and surgery Non hybrid group: surgery	In-hospital stay

*ISS*, Injury Severity Score; *CT*, computed tomography; *OT*, operating theatre; *IR*, interventional radiology

### Efficiency of the hybrid OT

Table 2 displays the various efficiency outcomes assessed by all studies. One important marker of efficacy of the hybrid OT, the transportation time from ED to first intervention, was assessed in 3 studies. Only Carver et al. showed a significant reduction in time compared to pre-RAPTOR suite patients suggesting early mobilisation of the trauma team, anaesthesia and IR staff may improve timeliness to first intervention (OT/IR) in multi-injured patients [6]. Conversely, Jang et al. stated that delayed time to first intervention for hybrid OT unstable patients was a result of prolonged resuscitation in the ED prior to transfer [10].

**Table 2:** Efficiency outcomes of the hybrid operating theatre (OT) as reported in the five included studies

Author, year	Reported outcomes			
	time from ER arrival to intervention	Procedure duration	Mortality	Blood transfusion requirements
Gross, 2009	Similar mean time to first operation between hybrid OT group (155 min) versus conventional group (187 min) [*P* = 0.35]	*Not assessed*	Similar 30-day mortality between hybrid OT group (17%) versus conventional group (22%) [*P* = 0.42]	*Not assessed*
Loftus, 2020	*Not assessed*	Similar median total procedure times in the hybrid OT group (135 min) versus conventional group (133 min) [*P* = 0.971]	Similar in-hospital mortality in the hybrid OT group (13%) versus conventional group (10%) [*P* = 0.579]	Decreased median red blood cell (0 vs 1; *P* = 0.001) and plasma (0 vs 1; *P* < 0.001) transfusion associated with the hybrid group 4–24 h after arrival
Carver, 2020	Shorter time to intervention in the hybrid OT group (82 min), compared to time to angiography (148 min) and time to OT (101 min) in the conventional group [*P* < 0.05]	Shorter procedure times in the hybrid OT group	Similar in-hospital mortality in the hybrid OT group (14%) versus conventional group (15%)	Lower blood transfusion rate in the hybrid OT group (25%) versus conventional group (16%) [*P* = 0.04]
Kataoka, 2016	*Not assessed*	Shorter total procedure time in the hybrid OT group (229 min) versus conventional group (335 min) [*P* = 0.007]	Similar in-hospital mortality in the hybrid OT group (15%) versus conventional group (36%) [*P* = 0.31]	No significant difference in mean blood transfusion volume during procedure between the hybrid OT group (4174 ml) and conventional OT group (5832 ml) [*P* = 0.24]
Jang, 2020	Similar median time to intervention between hybrid OT group (80 min) versus conventional group (75 min) *	*Not assessed*	*Not assessed*	*Not assessed*
	Median time from ED to OT arrival was shorter in conventional group			
	Median time from OT arrival to start of intervention was shorter in hybrid OT group			
	Within the hybrid OT group, time to intervention was longer for hemodynamically unstable patients compared to stable patients			

*Based on descriptive statistics, no statistical significance

The intraoperative duration was determined by whether patients underwent IR procedures solely or combined IR and concurrent surgery. Kataoka et al. showed a significant reduction in total intraoperative time of 126 min in their analysis of 13 hybrid OT patients [9]. In transferring patients to the MIGTS suite, Gross et al. showed overall fewer transfers of patients between ED and ICU [7]. In addition, all studies reported no statistically significant difference in mortality associated with the hybrid OT compared to the conventional OT group. In their RAPTOR suite, Carver et al. revealed that in a subgroup of hemodynamically unstable patients, those who underwent procedures in the hybrid OT were associated with a decrease in mortality [6]. This suggests that the hybrid OT setup may selectively benefit those most severely injured patients for whom rapid intervention by either IR or surgery to prevent exsanguination are most likely to benefit.

In addition, the impact of the hybrid OT on other secondary outcomes (blood transfusion requirements and in-hospital complications) was suggestive of improvement but undermined by the lack of consistent data reported across all studies [6, 8, 9]. Furthermore, only one study revealed fewer infectious complications, ventilator days and Clavien-Dindo type 4B complications associated with the hybrid OT group [8].

### Quality assessment

In terms of methodological quality, all studies were generally of high quality and low risk of bias (Table 3). Failure to control for type of intervention to ensure comparability of cohorts was a weakness identified in the study by Jang et al., as it attempted to compare patients who underwent angiographic procedures or combined angiographic and surgical procedures in the hybrid OT group against those who only underwent surgery in the conventional OT group. For follow-up duration, we deemed 30-day mortality as the best representation of trauma outcomes therefore weakness was identified in 4 out of 5 studies which assessed in-hospital mortality. Most studies received either no funding, or were funded by medical institutions or non-profit organizations.

**Table 3:** Risk of bias evaluation; A-I represents rating categories according to Newcastle–Ottawa Scale for assessing the quality of non-randomized studies

Study	A	B	C	D	E	F	G	H	I
Gross, 2009	✓	✓	✓	✓	✓	✓	✓	✓	✓
Loftus, 2020	✓	✓	✓	✓	✓	✓	✓	×	✓
Carver, 2018	✓	✓	✓	✓	✓	✓	✓	×	✓
Kataoka, 2016	✓	✓	✓	✓	✓	✓	✓	×	✓
Jang, 2020	✓	×	✓	✓	×	✓	✓	×	✓

A, Representativeness of the exposed cohort; B, Selection of the non-exposed cohort; C, Ascertainment of Exposure; D, Outcome of interest was not present at start of study; E, Comparability of cohorts- major factor controlled for; F, Comparability of cohorts- any additional factor controlled for; G, Assessment of outcome; H, Follow-up duration; I, Follow-up Adequacy

## Discussion

In managing traumatic injuries, the timeliness to definitive haemorrhage control remains a significant challenge to prevent mortality [3, 11]. A hybrid OT setup, armed with OT and IR capabilities, could potentially reduce the time interval from injury presentation to life-saving intervention [6]. However, its tremendous potential identified in 2013 has still not eventuated, limited by a paucity of available clinical data and a cost profile far beyond the typical public hospital budget [4].

The results of this systematic review highlight the limited impact of the hybrid OT in the context of severe trauma given the small study cohorts and lack of consistent outcome reporting. Contrary to expectation, the time from admission to first intervention and overall mortality were not consistently reduced or reported across the five studies. Only one study indicated the hybrid OT may enhance survival for a subgroup of hemodynamically unstable patients [6]. Common impediments encountered in transferring patients to the hybrid suite such as duration of initial ED resuscitation, time to CT scan and intra-hospital transfers were not consistently reported and are necessary to understand how further optimisations can be made. For instance, the MIGTS despite having an in-built CT scanner only reduced time to CT by 13 min, and the RAPTOR setup highlighted 40% of unstable cases undergoing CT scan prior to OT [6, 7]. Since few jurisdictions around the world could consider the MIGTS option or transfer unstable trauma patients to the CT scanner, the generalisability of the hybrid OT setup as described is very limited.

Identifying which patients may benefit from the hybrid OT was suggested as those that require a combination of percutaneous and open procedures [6]. Selecting those patients in the ED can be difficult but if the suspicion is high for non-compressible torso haemorrhage, combination of severe pelvic fractures and thoraco-abdominal injuries or obvious unstable penetrating trauma, then a direct transfer to the hybrid OT would be feasible. However, such a cohort of patients represented 7% of the total operative group [6], and 13 patients over 14 years only [9]. Furthermore, the time spent post intervention within the hybrid OT should also be reported as a rate limiting step to definitive ICU care.

The studies analysed did highlight the challenges of acquiring trained personnel to fully operationalise a hybrid OT [7, 10]. An available team on standby 24/7 comprising surgical, anaesthesia, IR, as well as OT technicians and scrub nurses is a complicated logistical exercise, particularly after-hours and on weekends as highlighted by Kataoka et al. [9] Others identified that the hybrid OT is a shared resource further limiting its availability for unplanned major trauma presentations [7]. Furthermore, the teams will need to overcome a steep learning curve and the subsequent time and costs for training. In the author’s own institution, the hybrid OT is a shared space with other disciplines such as vascular and transplantation. Anecdotally the IR clinicians have preferred to perform percutaneous procedures within their own department hence the establishment of a protocol with universal agreement is necessary before a hybrid OT setup can be fully operational. In one study, trauma surgeons were trained to perform common trauma IR procedures independently thus reducing reliance on the need for an on-site radiologist when operating within the hybrid ED [12].

The hybrid OT ‘one stop facility’ after ED resuscitation is not the only significant intervention designed to reduce time to definitive haemorrhage control. Direct to OT trauma resuscitation protocols bypassing the ED, with robust prehospital triaging systems, have already been adopted by various institutions to improve quality of care [13, 14]. In a study of more than 4000 patients over a 9-year period, Fischer et al. demonstrated the benefits of direct transfer to OT for severe trauma patients in terms of reduction in morbidity and mortality and increase in cost effectiveness [15]. A recent advance has also included the hybrid ED facility. Several trauma centres in Japan have proposed a novel trauma workflow using an integrated hybrid ED model, akin to a one-stop hybrid OT with CT scan capabilities within the ED, where severely injured trauma patients are directly admitted for resuscitation [12, 16, 17]. While the feasibility and costs of such a hybrid ED system have not been well established, early evidence has shown that it is associated with timeliness to intervention and some mortality benefit in severe trauma [12, 16, 17]. The findings of these studies have been summarized in Table 4.

**Table 4:** Summary of series’ comparing hybrid emergency department (ED) versus the conventional trauma workflow without a hybrid ED

Author, Institution, Year	Hybrid ED	Conventional	Baseline features (Hybrid ED versus Conventional)	Outcomes (Hybrid ED versus Conventional)	Remarks
Wada, OGMC, 2012	21	27	No significant difference	Shorter time to CT initiation and end of CT Shorter time to start of bleeding control procedures No significant difference in 28-day mortality	1 patient in the hybrid group and 7 patients in the conventional group were transferred to the OT for emergency surgery
Kinoshita, OGMC, 2019	336	360	Difference in mechanism of injury (fewer motor vehicle accidents) Higher prothrombin time international normalized ratio	Shorter time to CT initiation Shorter time to emergency procedure Decreased 28-day mortality Reduced number of deaths from exsanguination	Outcomes confirmed with propensity score analyses
Ito, TUSM, 2020	24	72	Younger age Greater proportion of patients with traumatic brain injuries, Glasgow Coma scale of < / = 8 and intubated on admission Lower Revised Trauma Score More frequent REBOA insertion and simultaneous or subsequent laparotomy/thoracotomy More frequent massive transfusion protocol activation	No difference in time from arrival to CT scan Shorter time from arrival to angioembolization No differences in rates of angioembolization complications, infectious complications and in-hospital mortality	Evaluated all patients who underwent angioembolization for pelvic fracture

OGMC Osaka General Medical Center, Japan; TUSM Teikyo University School of Medicine, Japan

Although the results of our systematic review were derived from the best available evidence, there are several limitations. Firstly, these are mostly observational cohort studies of small sample size, therefore selection bias may exist to limit the generalisability of the findings. In addition, as most studies comprised patients treated over a long time period (1997–2020), factors such as improved surgical or radiological expertise, changes in hospital protocols and evolving practices may impact the outcomes reported. Moreover, as both patients and trauma team members were not blinded in these studies, a preference towards hybrid OT may have contributed to increased efficiency and outcomes observed in the hybrid OT group. In our opinion, future studies should present results according to broad categories: identifying the sub-group with clear clinical benefit from the hybrid suite, efficiency targets such as transportation time, transfers between ED arrival and final ICU admission, direct clinical outcomes such as 30-day morbidity and mortality and a cost–benefit analysis. Realistically, this can only be achieved in a high-volume centre and preferably in a clinical trial setting. One may consider a multi-centre study between an established hybrid OT setup versus a traditional high-volume centre.

## Conclusion

Despite the findings of this systematic review, considerable time, effort and financial resources have been spent implementing the hybrid OT with the altruistic goal of preventing unnecessary deaths from major trauma and each jurisdiction must be commended for that. Being able to shave minutes from the ED assessment to definitive procedure is a multi-faceted dilemma and should be aggressively pursued. As mentioned, future studies should consider uniform endpoints and the possibility of a clinical trial. Until then any institution that is utilising a hybrid OT setup is encouraged to report their findings and add to the limited available data.

## Abbreviations

OT: Operating theatre
IR: Interventional radiology
CT: Computed tomography
ED: Emergency department

## Declarations

### Availability of data and materials

All data generated or analysed during this study are included in this published article.
